# Capsaicin-enriched diet ameliorates autoimmune neuritis in rats

**DOI:** 10.1186/s12974-018-1165-x

**Published:** 2018-04-24

**Authors:** Jeremias Motte, Björn Ambrosius, Thomas Grüter, Hussein Bachir, Melissa Sgodzai, Xiomara Pedreiturria, Kalliopi Pitarokoili, Ralf Gold

**Affiliations:** Department of Neurology, Ruhr University Bochum, St. Josef- Hospital, Gudrunstrasse 56, 44791 Bochum, Germany

**Keywords:** Chronic inflammatory demyelinating neuropathy (CIDP), Guillain-Barré syndrome (GBS), Capsaicin, Immune system, EAN, Experimental autoimmune neuritis

## Abstract

**Background:**

Autoimmune neuropathies are common PNS disorders and effective treatment is challenging. Environmental influence and dietary components are known to affect the course of autoimmune diseases. Capsaicin as pungent component of chili-peppers is common in human nutrition. An influence of capsaicin on autoimmune diseases has been postulated.

**Methods:**

We tested capsaicin in the animal model of experimental autoimmune neuritis (EAN) in Lewis rat. Rats were immunized with P2-peptide and were treated with capsaicin in different preventive settings. Electrophysiological, histological, and molecular biological analyses of the sciatic nerve were performed to analyze T-cell and macrophage cell count, TRPV1, and cytokine expression. Moreover, FACS analyses including the intestinal immune system were executed.

**Results:**

We observed an immunomodulatory effect of an early preventive diet-concept, where a physiological dosage of oral capsaicin was given 10 days before immunization in EAN. A reduced inflammation of the sciatic nerve was significant detectable clinically, electrophysiologically (CMAPs reduced in control group *p* < 0.01; increase of nerve conduction blocks in control group *p* < 0.05), histologically (significant reduction of T-cells, macrophages and demyelination), and at cytokine level. In contrast, this therapeutic effect was missing with capsaicin given from the day of immunization onwards. As possible underlying mechanism, we were able to show changes in the expression of the capsaicin receptor in the sciatic nerve and the small intestine, as well as altered immune cell populations in the small intestine.

**Conclusion:**

This is the first report about the immunomodulatory effect of the common nutrient, capsaicin, in an experimental model for autoimmune neuropathies.

**Electronic supplementary material:**

The online version of this article (10.1186/s12974-018-1165-x) contains supplementary material, which is available to authorized users.

## Background

The Guillain-Barré syndrome (GBS) is the prototype of acute demyelinating immune-mediated diseases in the PNS. The incidence of GBS ranges from 0.62 to 2.66 per 100,000 in North America and Europe [1, 2].

The chronic inflammatory demyelinating polyradiculoneuropathy (CIDP) is the most common chronic acquired demyelinating polyneuropathy with a prevalence of 1–8.9 cases per 100,000 people [3]. The classical CIDP is characterized by selective involvement of the peripheral nervous system, proximal as well as distal weakness and typically the involvement of both motor and sensory fibers. The course of disease has been described as monophasic with full recovering and no relapses, a relapsing–remitting course and a chronic progressive course associated with a progressive disability of the patients [4].

The first line therapies for CIDP are intravenous immunoglobulins, corticosteroids, and plasma exchange. Still, 25% of the patients do not respond to first-line treatment. Other highly effective immunosuppressive agents or even autologous bone marrow ablation have been reported as successful [5, 6]. Fifty percent of the patients suffer from clinical relapses after treatment [7] and show signs of a progressive demyelination and disability.

This clearly not only indicates the need to broaden the spectrum of therapeutic options for neuritis patients but also strategies of primary prevention.

The role of the intestinal immune system in the initiation and maintenance of autoimmune diseases has been increasingly studied in recent years [8–11], and the influence of the intestinal immune system in GBS is undisputed. Preceding gastrointestinal infections most frequently with *Campylobacter jejuni* (CJ) have been described as the most common trigger factor for GBS [12]. Antibodies against CJ are found in the serum of GBS patients implicating an intestinal mediated immune dysregulation [2]. Moreover, nutrition has been described to influence autoimmune diseases of the CNS [13–15]. So far, no relevant reports for diseases of the PNS were published. Though difficult to evaluate, a comparison of the GBS incidence rates suggest that incidence in Europe and North America is lower than in China, Brazil, Africa, Australia, and Japan [16], countries where the consumption of spicy food is traditionally higher [17].

This suggests that nutrition, especially spicy diet, may play a major role in the development of autoimmune polyneuropathies, maybe through influencing the intestinal immune system.

Capsaicin with the chemical structure (E)-N-[(4-hydroxy-3-methoxyphenyl) methyl]-8-methylnon-6-enamide is a hydrophobic alkaloid produced by peppers (Capsicum species; *Solanaceae*) and is responsible for the sharp taste of the genus [18]. The plant originates from Central and South America and was cultivated especially in Africa, south east Asia, and Japan. Until now, chili peppers are an integral part of local diet in these regions and its consumption varies worldwide interculturally as well as interindividually.

Capsaicin is the direct agonist of the transient receptor potential channel vanilloid subfamily member 1 (TRPV1) [19]. The TRPV1 channel is a non-selective cation channel, with a wide expression in different cell types of the nervous system as well as the immune system (neurons, glia cells, monocytes) [20–28]. Moreover, the TRPV1 receptor was described in different parts of the small intestine [8, 22, 29–35].

Its suspected biological function is to detect noxious changes of the cell environment (pH, temperature, mechanical stress) [36]. TRPV1 was described as potential target in autoimmune diseases in generally [8, 37], as well as a modulator of neuroinflammation in particular [28]. The TRPV1 receptor is a target for direct immunomodulatory pathways in immune cells, especially on regulatory macrophages [8, 27, 34]. Recently, the regulatory phenotype of CD11b^+^F4/80^+^-macrophages in the mesenterial lymph nodes was described in an autoimmune diabetes mouse model after treatment with oral capsaicin [8]. In addition, this receptor is also known as part of indirectly immunomodulatory pathways when expressed in unmyelinated nerve fibers. These cells contain the neuropeptide calcitonin gene-related peptide (CGRP), which has a key role in the neuroimmune axis by influencing T/B lymphocytes, dendritic cells, mast cells, and macrophages [38, 39]. In CD4^+^ T-cells, CGRP induces elevation of cellular cAMP levels and inhibits production of TNF-alpha, IFN-gamma, and NFkB. Also a positive effect of the release of IL-4 was described [40]. The biological effects differ depending on the dose, application form, and the bioavailability of capsaicin [41, 42].

A commonly used animal model to investigate demyelinating peripheral neuropathies is the experimental autoimmune neuritis (EAN) in Lewis rats, which is induced by peripheral nervous system (PNS) antigens, e.g., P2 [43–45]*.* EAN simulates electrophysiological and histological characteristics as well as many aspects of the humoral and cellular immunoresponse of GBS and CIDP [46–49]. The aim of this study was to determine the role of capsaicin in EAN rats in a concentration equal to common spicy food.

We present the first report of an immunomodulatory effect of preventive, oral capsaicin therapy in the animal model of peripheral neuropathies.

## Methods

### Induction of EAN and assessment of clinical score

The present study was carried out in accordance with the European Communities Council Directive of 22 September 2010 (2010/63/EEC) for care of laboratory animals and after approval of the local government ethics committee (Bezirksamt, Arnsberg, Az.: 84-02.04.2016.A333).

In the presented study, a total of 92 female Lewis rats (65 treated with different concentrations of capsaicin, 27 in control groups, 6 independent experiments) with an age of 6–8 weeks were purchased from Charles River (Sulzfeld, Germany). The animals were in a range of weight 160–180 g when treatment started and at least 1 week in the animal facility, to get accustomed to the new environment. Rats were housed under standardized, pathogen-free conditions at the local animal facility (Medical Faculty, Ruhr-University Bochum, Bochum) where food and water were given ad libitum*.* The rats were randomly divided into control and treatment groups. The end of the experiment was either day 16 (peak of disease) or day 23 (recovery phase) post immunization (p.i.) (Additional file 1: Figure S1).

Two principle different groups were investigated (Additional file 1: Figure S1):

- *Early preventive diet concept:* Animals were pre-treated with capsaicin for 10 days before immunization (day − 9 p.i.). After immunization capsaicin therapy was continued (5 independent experiments, *n* = 76).
- *Late preventive diet concept:* Animals were immunized (day 0 p.i.), and therapy with capsaicin started at the day of immunization (one experiment, *n* = 16).

After 10 days of pre-treatment or on day 0 without pre-treatment, rats were immunized by subcutaneous injection of 250 μg P2_53-78_ into the tail base. The neuritogenic P2 peptide, corresponding to the amino acids 53–78 of rat myelin P2 protein, was synthesized by Dr. Rudolf Volkmer from Charité–Universitätsmedizin Berlin (Berlin, Germany). P2 was emulsified in equal volume of complete Freund’s adjuvant (CFA), containing 1 mg/ml *Mycobacterium tuberculosis* H37RA (Difco, Detroit, USA). Meanwhile, they were anesthetized by exposure to 1.5–2.0% halothane in oxygen, eyes well-protected against desiccation (Dexpanthenol, Bayer Vital GmbH, Leverkusen, Germany). The animals were scored and weighted two times before immunization in early preventive diet concept group. With start of immunization the body weight and symptoms were assessed daily. The determination of the disease score was based on a 10-fold system (0 normal; 1 less lively; 2 impaired righting/limb tail; 3 absent righting; 4 ataxic gait, abnormal position; 5 mild paraparesis; 6 moderate paraparesis; 7 severe paraplegia; 8 tetraparesis; 9 moribund; 10 death) (Enders et al. 1998).

### Capsaicin for in vivo treatment

Capsaicin, synthesized from Alps Pharma, as a 93.1% pure powder, was used for all experiments. It was dissolved in rapeseed oil (Fauser Vitaquell, Hamburg, Germany). Once daily, rats received 200 μl rapeseed oil with different concentrations of capsaicin by oral gavage. The tested concentrations of capsaicin ranged from 0.01 to 10 mg/kg capsaicin daily. The control-group was treated with 200 μl pure rapeseed oil.

### Electrophysiological analysis

Nerve conduction tests were performed by a blinded investigator 1 day before immunization (day − 1 p.i.) and at the end of the experiment at day 16 p.i. (maximum of natural disease course) or day 23 p.i. (natural recovery phase) as described earlier [50].

In short, animals were anesthetized intraperitoneally (i.p.) with 10 mg/kg Xylazine (Xylavet, CP-Pharma, Burgdorf, Germany) and 50 mg/kg Ketamine (CP-Pharma, Burgdorf, Germany). A fully digital recording Keypoint apparatus (Dante, Skovlunde, Denmark) was used in combination of paired needle electrodes, which were inserted into the sciatic notch (hip, proximal) and into the popliteal fossa (knee, distal). The sciatic nerve was stimulated with supramaximal rectangular pulses of 0.05 ms duration, and the resulting compound muscle action potential (CMAP) was recorded from needle electrodes placed subcutaneously over the dorsal foot muscles. A ground electrode was placed between the distal stimulating electrode and the active recording electrode. To calculate the motor nerve conduction velocity (MNCV), the distance between stimulating cathodes was divided by the difference of the latency. Similarly, the persistence and minimum latency of 10 F-waves evoked by stimulation at the popliteal fossa were recorded for the right side (Tuck et al. 1982, Taylor et al. 2003). Temperature differences were minimized by conducting the study as soon as the anesthesia had taken effect and by warming the leg with a heating lamp.

### Histopathological assessment and immunohistochemistry

Transcardial perfusion with PBS (ThermoFisher, Schwerte, Germany) was performed on disease maximum (day 16 p.i.) or in recovery (day 23 p.i.). The right sciatic nerves were dissected and embedded in Neg-50 (ThermoFisher, Schwerte, Germany), snap frozen and stored at − 80 °C. Moreover, the small intestine was obtained, fixed in paraformaldehyde (PFA; 4%) over 24 h and dehydrated by sucrose solution (30%). Following, it was also embedded in Neg-50 and snap frozen. For histopathological assessment, the tissue was sectioned (8 μm) on a cryostat (ThermoFisher, Schwerte, Germany) and was mounted on deep frozen approved glass slides (Hartenstein, Würzburg, Germany).

Immunohistological staining were performed using the DAKO animal research kit for primary mouse antibodies (Dako, Hamburg, Germany) as described by the manufacturer’s protocol. Monoclonal antibodies against T-cells (Pan T-Cells, 1:100, Hycultec, Beutelsbach, Germany) and macrophages (ED1, CD68, 1:100 Hycultec, Beutelsbach, Germany) were used.

Stained tissue was microscope (BX51; Olympus, Tokyo, Japan) equipped with an Olympus DP50 digital camera. Images (× 20 magnification) of 8 transverse sections of the sciatic nerve from each animal were digitally generated (Cell^F 5.1, Olympus, Tokyo, Japan). Stained cells were counted in ImageJ (National institutes of Health, Bethesda, USA). Counts were multiplied by 10.47468878 to archive cells per square millimeter tissue.

For identification of TRPV1, S-100, CD3, and CD68, the following antibodies were used: TRPV1-antibody (1:1000 ThermoFisher, Schwerte, Germany), anti-S-100 antibody (1:100, Merck Millipore, Darmstadt, Germany), CD3-antibody (1:100 Invitrogen ThermoFisher, Schwerte, Germany), and CD68-antibody (1:100 Hycultec, Beutelsbach, Germany). Secondary antibodies conjugated with Alexa 488 and Alexa 555 (1:1000) (ThermoFisher, Schwerte, Germany) were used according to manufacturer’ s protocol and DAPI-Fluoromount (4′,6′ diamino-2-phenylindole 2HCl, Biozol, Eching, Germany) was used for nuclear fluorescent staining of DNA.

For assessment of demyelination, lesions were identified by the accumulation of nuclei and absence of FluoroMyelin™ Red fluorescent stain (1:300, ThermoFisher, Schwerte, Germany) performed according to the manufacturer’s protocol.

Fluorescent signals were detected using an inverted fluorescence microscope (BX51; Olympus, Tokyo, Japan) equipped with an Olympus DP50 digital camera. For assessment of fluorescent staining, images (× 20 magnification) of 8 transverse sections of the sciatic nerve from each animal were digitally generated (Cell^F 5.1, Olympus, Tokyo, Japan). The percentage of the area of TRPV1, CD3, or CD68 stained cells or the cell count of co-stained cells per section was determined using image analysis software ImageJ (National institutes of Health, Bethesda, USA). Omission of the primary antibodies served as negative control.

### Tissue preparation, RNA isolation, and gene expression analyses with quantitative RT-PCR

Total RNA was isolated from the left sciatic nerve or small intestine samples of rats either on disease maximum (day 16 p.i.) or in recovery (day 23 p.i.) using the RNeasy Mini extraction kit (Qiagen, Hilden, Germany). At 37 °C overnight, all samples were treated with the RNA Stabilization Reagent (RNAlater, Qiagen, Hilden, Germany) and were stored at − 80 °C until use. According to the manufacturer’s protocol, total RNA was reverse-transcribed into cDNA for the Reverse Transcription System (Promega, Madison, WI, USA). Messenger-RNA expression levels were analyzed for IFN-gamma, TNF-alpha, IL-10, IL-4, F4/80, FoxP3, TRPV1, and CGRP by quantitative RT-PCR according to the manufacturer’s instructions (Applied Biosystems, Foster City, CA, USA). Sequence-specific primers and probes were designed using pre-developed GoTaq® assay reagents (Promega, Madison, WI, USA). RT-PCR amplifications were carried out using the real-time PCR System 7500 (Applied Biosystems). Real-time PCR for RNA-to-CTTM 1-Step was performed according to the following amplification protocol: 25 °C for 5 min, 42 °C for 30 min and 82 °C for 5 min (for cDNA synthesis), 95 °C for 3 min (transcriptase inactivation) followed by 40 cycles of 95 °C for 15 s, and 60 °C for 1 min. Beta-Actin and Glyceraldehyde 3-phosphate dehydrogenase (GAPDH) were used to normalize mRNA expression. To compare each mRNA and housekeeping β-Actin as well as GAPDH mRNA expression, the relative expression of PCR products was determined using the ΔΔCt method (Schmittgen and Livak, 2008). Each experiment was performed in duplicate and the mean Ct was used in the equation.

### Isolation of mononuclear cells and FACS analyses

On day 16 or 23 p.i., blood samples obtained by cardiac puncture as well as spleen were obtained before perfusion, inguinal lymph nodes were removed after transcardial perfusion with PBS (ThermoFisher, Schwerte, Germany). Peyer’s patches (PP) and intestinal lymph nodes were prepared from small intestine under aseptic conditions. Erythrocytes were lysed using ACK buffer (150 mM NH_4_Cl, 10 mM KHCO_3_, 0,1 mM Na_2_EDTA). Single cell suspensions of mononuclear cells (MNC) from individual rats were prepared separately. Cells were washed twice in PBS and stained with monoclonal antibodies. We evaluated the frequency of CD8^+^ T-cells, CD4^+^ T-cells, CD11b^+^ monocytes, CD4^+^CD11b^+^ dendritic cells (DCs), CD4^+^ CD25^+^ FoxP3^+^ regulatory T-cells (Tregs), and CD4^+^CD11b^−^MHCII^+^ plasmacytoid DCs by fluorescence-activated cell sorting (FACS) staining (eBioscience, San Diego, CA, USA) in accordance with the manufacturer’s instructions. Intracellular staining for FoxP3 was performed using the FoxP3 Staining Set (eBioscience) according to the manufacturer’s instructions. Flow cytometry were performed with a FACS Canto II (BD Pharmingen, Heidelberg, Germany) and DIVA Software (BD Pharmingen, Heidelberg, Germany).

### Statistical analysis

Statistical analyses were performed using Graph Pad Prism 6 (GraphPad Software Inc., San Diego, USA). Area under the curve (AUC) was calculated for clinical courses and analyzed by non-parametric Kruskal–Wallis one-way analysis of variance. Multi comparison was performed for clinical courses and analyzed by two-way ANOVA, Dunnett’s test multiple comparison test. Histological, electrophysiological and flow cytometry experiments were also compared using ANOVA combined with Tukey’s multiple comparisons test. Chi-square test was used to analyze categorical outcomes (incidence and onset of disease). Differences between pairs of groups were tested by Student’s *t* test. Data is presented as mean ± SEM or as mean ± SD. Probability level (*p* value) are indicated as *p ≤ 0.05, **p ≤ 0.01, and ***p ≤ 0.001.

For histological analysis, slides were blinded by a not-involved third person and labeled with a numeric-code, which was unblinded after analysis.

## Results

### Long-term diet of capsaicin attenuates the clinical course of experimental autoimmune neuritis

Two groups were investigated:

- An early preventive diet, receiving capsaicin 10 days before immunization and continued after immunization
- A late preventive diet, receiving capsaicin from day 0.

**Fig. 1:**
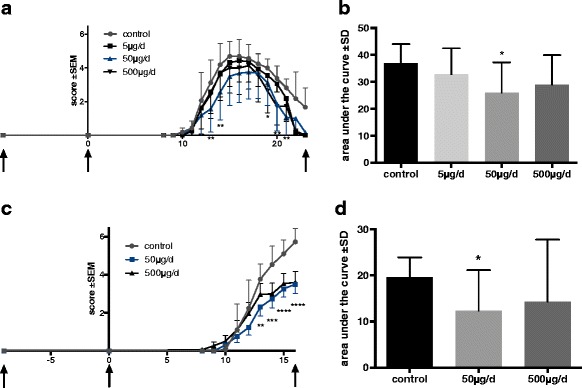
Clinical EAN course under early preventive oral capsaicin treatment. EAN was induced in Lewis rats by immunization on day 0 (second arrow) with P2 peptide 53–78 plus CFA. Rats received capsaicin diluted in rapeseed oil at doses of 5, 50, and 500 μg/d once daily from day − 10 (first arrow) to day 23 (third arrow) p.i. (*n* = 31; pooled data from two independent experiments, (**a**, **b**) or doses of 50 and 500 μg/d to day 16 p.i. (*n* = 45; pooled data from three independent experiments, (**c**, **d**) by oral gavage. Control rats received always rapeseed oil only. Mean values of disease score ± SEM; area under curve (AUC) ± SD are depicted; two-way ANOVA multiple comparison test; ANOVA Kruskal–Wallis test

Rats were immunized with peptide P_53-78_ on day 0. Daily oral gavage of capsaicin dissolved in rapeseed oil started depending of the stratification at day − 9 p.i. or day 0 p.i.. Clinical signs of EAN occurred around day 8-10 p.i. and progressed till day 16-18 p.i., before clinical recovery started. Treatment of the rats with dosages of 50 μg/d and 500 μg/d in an early preventive diet concept, ameliorated the clinical EAN course and caused significantly less clinical signs compared to control animals (*p* < 0.001, two-way ANOVA, Dunnett’s test multiple comparison test, *p* < 0.05 AUC one-way ANOVA, Kruskal–Wallis test) (Fig. 1). The greatest clinical effect was observed in the 50 μg/d group. In contrast, animals of late preventive diet group did not benefit significantly from capsaicin in these dosages (data not shown). However, higher and unphysiological concentrations up to 10 mg/kg (1200 μg/d) capsaicin in the late preventive concept did show a significant clinical effect (data not shown).

The following results describe the effect in the early preventive diet group only.

Dosages of 5 and 500 μg/d capsaicin caused a less strong effect of clinical signs compared to the 50 μg/d group (Fig. 1, five independent experiments, with three to five animals per group). The EAN incidence in control group was 100%, in group 50 μg/d 89% ±15%, in group 500 μg/d 96% ± 9%. Median day of disease onset was day 9.8 ± 0.58 in control group, in group 50 μg/d day 10.6 ± 1.73 and in group 500 μg/d day 9.8 ± 0.96. All differences not reaching significance levels in chi-square test (values ± SD).

No significant body weight reduction or further toxic effects of capsaicin at any dosage were found.

### Capsaicin improves proximal and distal nerve conduction

Electrophysiological measurements of the sciatic nerve at different stages of the clinical course were performed and following measures were evaluated (Additional file 2: Figure S2):

1. Demyelination, characterized by the reduction of motor nerve conduction velocity (MNCV)
2. Degree of focal demyelination, as implied by conduction block (conduction block was defined as a 50% reduction of the amplitude after proximal stimulation without significant dispersion relative to the distal CMAP) and axonal damage represented by mean CMAP
3. Lumbar root involvement, depicted by prolongation of F-wave latencies

In recovery phase of EAN (day 23 p.i.) the measurements of MNCV showed a higher nerve conduction velocity in the treated group (50 μg/d). At the maximum of disease there was no difference between the groups (Additional file 2: Figure S2A).

Further electrophysiological measurements of the sciatic nerve at the maximum of the clinical course (day 16 p.i.) showed a more than 50% reduction of the CMAP in the control group as an indicator of conduction block (mean CMAP on day − 1 p.i. 7.44 mV vs. day 16 p.i. 2.98 mV, *p* < 0.01, *n* = 15, *n* = 5 animals per group). In the control group four of five animals suffered from a conduction block defined as CMAP reduction of at least 50%. In the 50 μg/d group only one of five and in the 500 μg/d group zero of five animals showed a conduction block (*p* < 0.05, *n* = 15, *n* = 5 animals per group).

The animals treated with 50 and 500 μg/d did not show significant reduction of the average CMAP amplitude after proximal and distal stimulation. In recovery phase of the disease, this tendency is also present (Additional file 2: Figure S2 B). F-wave latency was significantly prolonged in control group whereas both treatment groups did not show a prolongation of F-waves (mean F-wave latency of control group on day − 1 p.i. 7.54 ms vs. day 16 p.i. 9.58 ms, *p* < 0.01, *n* = 15, *n* = 5 animals per group).

In recovery phase, the group treated with 50 μg/d also showed normal F-waves; however, the control group also showed normal values at this time (Additional file 2: Figure S2C).

### Capsaicin treatment reduces histological signs of demyelination as well as infiltration of T-cells and macrophages in the sciatic nerve

**Fig. 2:**
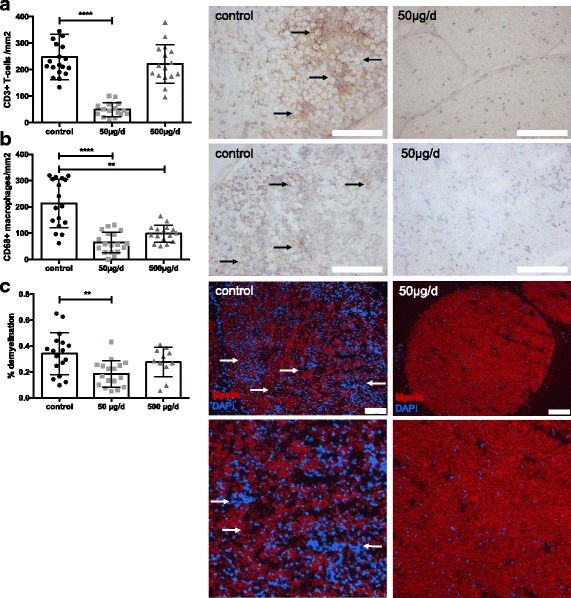
Capsaicin treatment reduced inflammatory infiltrates of T-cells and macrophages as well as demyelination in sciatic nerves of EAN rats. Rats were daily force fed with capsaicin or vehicle from day − 10 p.i. to day 16 p.i. (at expected disease maximum), sciatic nerves were isolated, and stained for CD3^+^ cells (**a**) and CD68^+^ cells (macrophages) (**b**). Representative photos of sciatic nerves in transverse sections of control animals and 50 μg/d capsaicin-treated animals. Scale bars indicate 100 μm. Mean numbers of T-cells per square milimeter sciatic nerve sections and mean numbers of macrophages (CD68^+^) per square milimeter sciatic nerve sections as calculated by immunohistochemistry on day 16 p.i. from EAN rats receiving capsaicin orally at different doses (50 and 500 μg/d) and control rats are shown in diagram placed in front of the images. Mean values and SD are depicted (**p < 0.005, ****p < 0.0001) (*n* = 10/group, data from two independent experiments). Fluoromyelin staining (**c**) shows reduction of demyelinated area. Mean values and SD were depicted (**p < 0.005, *n* = 8/group, data from two independent experiments ANOVA Kruskal–Wallis test). Scale bars indicate 100 μm, lower row 400 × 400 μm

We hypothesized that the improved clinical course of EAN goes along with a reduction in inflammatory infiltration of the PNS. T-cell infiltrates were significantly suppressed by capsaicin dosages of 50 μg/d (*p* < 0.001) at maximum disease course (day 16 p.i.) (Fig. 2a). In recovery phase of the disease (day 23 p.i.), T-cell infiltrates were also significantly suppressed in the group of 500 μg/d (*p* < 0.01) (data not shown). The effect of capsaicin suppressing inflammatory infiltration into the sciatic nerve was even stronger for macrophages at maximum of the disease (50 μg/d *p* < 0.0001; 500 μg/d *p* < 0.001) (Fig. 2b, c). In the recovery phase of the disease, the suppressing effect of macrophages was also significant in both groups of capsaicin treatment (50 μg/d *p* < 0.001; 500 μg/d *p* < 0.01) (data not shown).

Moreover, the reduction of infiltrating effector cells and electrophysiological signs of reduced proximal and distal demyelination came along with histological signs of more intact myelin. Figure 2 shows representative pictures of FluoroMyelin™ staining. Administration of 50 μg/d oral capsaicin in the long-term diet significantly reduced the percentage of demyelination of the sciatic nerve when compared to oil-treated control rats at maximum and recovery phase of the disease by 54% (Fig. 2c, *n* = 24, *p* < 0.05).

### Capsaicin reduces expression of pro-inflammatory cytokines and enhances expression of anti-inflammatory cytokines in the sciatic nerve

The clinical, electrophysiological, and histological results supported an immunomodulatory effect of oral capsaicin in EAN. To show inflammatory pathways addressed by capsaicin, the cytokine response pattern after capsaicin treatment was investigated in sciatic nerve.

**Fig. 3:**
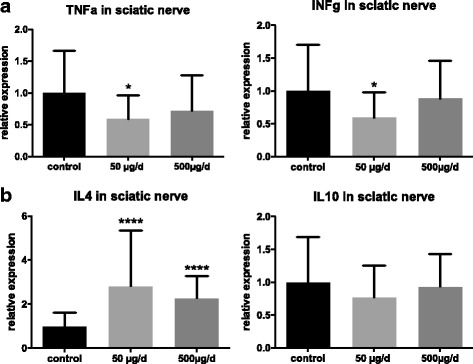
Pro-inflammatory cytokines were decreased, anti-inflammatory cytokine IL-4 was increased after oral capsaicin treatment. Rats were daily force fed with capsaicin or rapeseed oil from day − 10 p.i. to day 16 p.i. (at expected disease maximum), sciatic nerves were isolated and RT-PCR was performed for different cytokines. Pro-inflammatory cytokines TNFa and INFg were decreased (**a**), anti-inflammatory cytokine IL-4 was increased (**b**). Expression of IL-10 was not significant changed. Mean values and SD were depicted (*p < 0.05, ****p < 0.0001; *n* = 45, *n* = 15/group, pooled data from three independent experiments)

The balance between pro-inflammatory and anti-inflammatory cytokines (IFNg, TNFa, IL4) showed a strong anti-inflammatory pattern at the disease maximum (day 16 p.i.) in sciatic nerve after treatment with capsaicin (Fig. 3a, b).

The pro-inflammatory macrophages activating cytokines TNFa and IFNg were significantly reduced in the 50 μg group; in contrast, the anti-inflammatory and inducing class switching IL-4 was upregulated [51] (*p* = 0.05, *p* < 0.01, *p* < 0.001 *n* = 45, *n* = 15/group, pooled data from three independent experiments with similar results).

Moreover, the marker F4/80 for macrophages in sciatic nerve was significantly reduced in the 50 μg/d group in RT-PCR (*p* = 0.05, *n* = 30, *n* = 10/group, the experiment was repeated 2 times with similar results). Further analyses of expression of FoxP3 via RT-PCR in the sciatic nerve did not differ in either group (data not shown).

In the recovery phase of the disease (day 23 p.i.), the inflammatory response was already decreased and expression of these cytokines did not vary in RT-PCR.

### FACS analyses did not reveal influence on peripheral organs of the immune system

The FACS analyses of the spleen, blood, and inguinal lymph nodes did not show significant effects of the oral capsaicin treatment. There was only a slight tendency of CD11b^+^-cell reduction in blood during the peak of disease (data not shown).

### Capsaicin induces TRPV1 expression in sciatic nerve

**Fig. 4:**
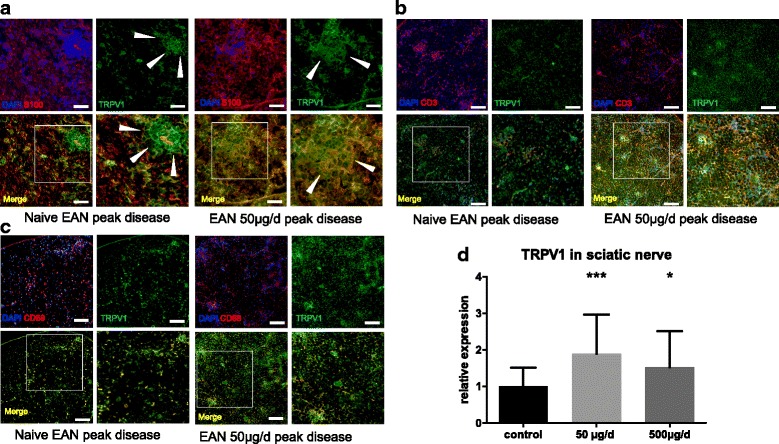
Expression of capsaicin receptor TRPV1 increased after treatment in sciatic nerve, co-expression was higher on macrophages than T-cells. For better comparison, sections were always selected in similarly infiltrated nerve sections. **a** Inflammatory infiltrate in sciatic nerve. Expression of TRPV1 in sciatic nerve (green) and S100 (red) as marker for Schwann cells in naïve EAN and EAN treated with capsaicin. The infiltrating cells express TRPV1 even in naïve EAN and displace Schwann cells (white arrows). After capsaicin treatment, infiltrating cells show a diffuse increase of TRPV1 (white arrows). **b** CD3+ T-cells (red) infiltrate sciatic nerve. In naïve EAN, the T-cell-infiltrates show low expression of TRPV1. After treatment with capsaicin T-cell-infiltrates show a distinct increase of TRPV1 expression (white arrows). **c** Co-expression of TRPV1 (green) in infiltrates of CD68+ macrophages (red) is obvious in naïve EAN as well as in capsaicin treated animals. After treatment TRPV1 expression increased. **d** Rats were daily force fed with capsaicin or rapeseed oil from day − 10 p.i. to day 16 p.i. (at expected disease maximum), sciatic nerves were isolated, and RT-PCR was performed for capsaicin receptor TRPV1. TRPV1 increased after capsaicin treatment in RT-PCR. Mean values and SD were depicted ((*p < 0.05, ***p < 0.001; *n =* 45, *n* = 15/group, pooled data from three independent experiments). Scale bars indicate 50 μm in figure (**a** 100 μm in Figure **b** and **c**)

We investigated TRPV1 expression in the peripheral nerve after treatment with capsaicin. At the peak of disease (day 16 p.i.), the TRPV1 expression increased significantly in the sciatic nerve after oral capsaicin treatment with 50 μg/d, respectively, 500 μg/d (Fig. 4a), whereas in remission of disease (day 23 p.i.), no difference of the expression of TRPV1 in RT-PCR was detected.

In histological sections, the TRPV1 receptor was expressed in the endoneurium, on T-cells, CD68^+^ cells, and on Schwann cells; however, the co-expression was different (Fig. 4b, c, d).

Our RT-PCR-assisted analysis of CGRP expression in the sciatic nerve, a suspected key neurotransmitter in the neuro-immune axis and involved in the TRPV1 pathway, did not show a significant increase in CGRP expression after capsaicin treatment between treated and untreated rats. However, a wide spread of the measured values was also evident with a likewise clear tendency for the CGRP decrease (*p* = 0.0681, *n* = 45 all experiments, experiments = 3, *n* = 15/group, Additional file 3: Figure S3A).

### 3.5 Oral capsaicin treatment could induce potentially regulatory macrophages in the small intestine

**Fig. 5:**
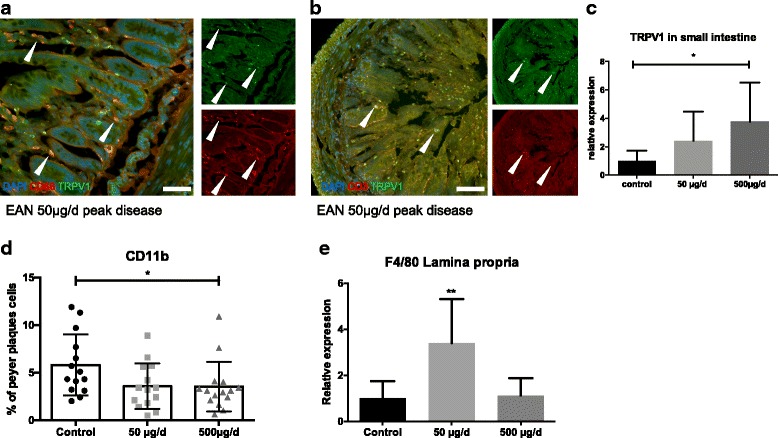
Expression of capsaicin receptor TRPV1 increased after treatment in small intestine, possible redistribution of immune cells in small intestine. **a** Immunohistological staining of TRPV1 and CD68 in small intestine revealed an expression of capsaicin receptor TRPV1 on CD68^+^ macrophages indicated by white arrows. **b** Immunohistological staining of TRPV1 and CD3 in the small intestine revealed also an expression of capsaicin receptor TRPV1 on CD3^+^ T-cells however in with lower co-expression (white arrows). **c** Rats were fed daily with capsaicin or rapeseed oil from day − 10 p.i. to day 16 p.i. (at expected disease maximum), the small intestine was isolated, and RT-PCR was performed for capsaicin receptor TRPV1. TRPV1 increased after treatment in RT-PCR. Mean values and SD were depicted (*p < 0.05, *n* = 15, *n* = 5/group). **d** FACS analyses showed a decrease of CD11b^+^ cell population in Peyer’s paches (*p < 0.05, *n* = 45, *n* = 15/group, pooled data from three independent experiments). **e** Expression of macrophage marker F4/80 showed an increase in lamina propria in RT-PCR (**p < 0.001, *n* = 15, *n* = 5/group)

Histological staining demonstrated TRPV1 expression on T-cells (CD3) as well as on macrophages (CD68) in small intestine (Fig. 5b). The expression of TRPV1 was upregulated after oral treatment with capsaicin in the lamina propria (*n* = 15, *n* = 5 animals per group) (Fig. 5a).

After oral capsaicin treatment, CD11b^+^ cells were reduced in PP in flow cytometry (*n* = 45, *n* = 15/group, pooled data from three independent experiments with similar results) (Fig. 5c), whereas F4/80-expression were increased in RT-PCR of the lamina propria compared to control (*n* = 15, *n* = 5 animals per group) in these animals (Fig. 5d). However, in FACS analysis and in RT-PCR, no differences in frequency or total numbers of CD4/CD25^+^ FoxP3^+^ in the PP after treatment were detected (Additional file 3: Figure S3B).

## Discussion

The aim of this study was to investigate the effect of orally administered capsaicin in human diet analogous dosages in EAN of Lewis rats.

Our results show that after a long-term diet concept (early preventive), receiving capsaicin ten days before immunization and continued after immunization food usual dosages result in amelioration of the disease course. This goes along with reduced T-cell and macrophage inflammation and a reduced demyelination in histology as well as signs of a reduced damage of axons reflected by electrophysiology.

Electrophysiological data give a clue for a reduced demyelination and a reduced involvement of spinal nerve roots in the phase of acute inflammation. Moreover, the mean values of CMAP could implicate a reduced secondary axonal damage.

Interestingly, representative concentrations of capsaicin (50 μg/d) in rats, corresponding to a consume of one to two chili-peppers per day in humans (converted to the body surface), showed the strongest effect in the treatment of EAN. This concentration revealed an improvement of the clinical score, inflammatory, and electrophysiological parameters in all experiments.

In contrast, in a late preventive concept, oral treatment with capsaicin from day 0 post immunization unfolds a therapeutic effect only after relatively (unphysiological) high dosages.

Our results are consistent with those reported by Nevius et al. who reported a clinical effect of capsaicin treatment in autoimmune diabetes mice in comparatively low dosages [8]. Capsaicin induces a strong reduction particular of CD68^+^ macrophages in rat peripheral nerves in the two effective dosages (50 and 500 μg/d), which represent a main inflammatory population in EAN. Moreover, capsaicin treatment results in a reduced demyelination in nerve conduction studies as well as in myelin staining. The clinical, electrophysiological, and histological effects were further supported by decreased pro-inflammatory and increased anti-inflammatory cytokines in the sciatic nerve. These results suggest a Th2 shift in immune response after treatment. Toxic effects of capsaicin were not observed in our experiments.

The TRPV1-receptor was described as capsaicin receptor in 1997 [19]. Its expression is reported among neurons of different compartments of the central and the peripheral nerves system like astrocytes, pericytes, in central neurons as well as in dorsal root ganglia (DRG), the trigeminal ganglion, and the murine plexus. Moreover, in many other cell types like glia cells, T-cells, macrophages, and the blood brain barrier [20–22, 28, 34, 36, 37, 52–55]. The most obvious assumption was that the therapeutic effect of capsaicin is mediated via TRPV1. Previous studies showed that capsaicin is absorbed from the stomach and the intestine rapidly by a passive process following oral administration [41]. Blood and intestine show the peak concentration of capsaicin 1 h after administration [56]. The bioavailability depends on the metabolism of capsaicin which differs depending on the tissue. For example, the half-life period of capsaicinoids is short in the liver, in contrast, long in the nervous system [41].

However, the site where capsaicin develops its therapeutic effect in EAN is not yet clear. Our study shows that the TRPV1-receptor is upregulated in RT-PCR of the sciatic nerve after oral capsaicin treatment in EAN. Our histological staining showed TRPV1 co-localized on infiltrating T-cells and macrophages. In Schwann cell culture, a constant expression of TRPV1 after capsaicin treatment was described recently [57]. That suggests an upregulation of TRPV1-receptor in infiltrating immune cells after treatment with capsaicin. The expression of the downstream molecule calcitonin gene-related peptide (CGRP) did not reveal significant effects after capsaicin treatment in RT-PCR of the whole sciatic nerve. Yet, we found a trend (*p* = 0.0681) to an increasing CGRP expression. Maybe the compartment “sciatic nerve” is too heterogenous to find distinct changes of CGRP expression. Further studies should investigate the individual parts of the sciatic nerve like Schwann cells, neurons, fibroblasts, T-cells, and macrophages to investigate this potential pathway.

As capsaicin was found in intestinal tissues, jejunum, and serosal fluid after oral ingestion in previous studies, we investigated the effect of capsaicin in the intestinal immune system [41, 42]. The role of the intestinal immune system in the initiation and maintenance of autoimmune diseases has been increasingly focused in recent years [8–10, 13]. These insights also guided the view on the small intestine beside the sciatic nerve itself in our case. The TRPV1 receptor was extensively described in the small intestine particularly in the myenteric plexus as well as on different immune cells itself [8, 22, 29–35]. Recently, Nevius et al. described a regulatory phenotype of CD11b^+^ F4/80^+^-macrophages in the pancreatic lymphnodes, which were induced after oral capsaicin treatment in a mouse model of autoimmune diabetes [8]. They showed that oral administration of capsaicin increases an immunomodulatory dendritic cell population in mesenterial lymph nodes, which attenuates the proliferation and activation of autoreactive T-cells in pancreatic lymph nodes and protects mice from development of autoimmune type 1 diabetes. The effect of capsaicin was not a result of Treg function. In agreement with these findings, there was no recognizable difference in CD4/CD25^+^ FoxP3^+^ regulatory T-cells in the PP, spleen, inguinal lymph nodes, and blood after capsaicin treatment.

Furthermore, we showed a decrease of CD11b^+^ cells in PP after oral capsaicin treatment in EAN, as well as an increase of F4/80-expression in RT-PCR of the lamina propria of the small intestine in these animals. This could be a redistribution of potential regulatory macrophages and is an interesting approach explaining the immunomodulating effect of capsaicin in EAN.

In contrast to the results from sciatic nerve, in the small intestine, the increase of F4/80 macrophages did not correlate proportionally with an increase of the TRPV1 receptor expression in the small intestine. This suggests a further TRPV1-independent effect of capsaicin which was described recently [58, 59] and should be investigated in further studies. The pathways of p38 MAPK and MAPKAPK2 (MK2) are particularly interesting, because of their expression in immune cells.

In our experiments, it remains unclear if the effect of intestinal immune system is TRPV1 driven; however, Nevius et al. described a TRPV1 receptor effect in small intestine. The differences in TRPV1 expression in sciatic nerve and small intestine in response to different capsaicin concentrations in treatment could be a result of the different bioavailability of capsaicin depending on the tissue as described above. In the sciatic nerve, the biological half-life is significantly longer compared to the small intestine [41]. This means a longer effect of capsaicin in the nervous tissue. Capsaicin is known to have a partly paradoxical effect on the receptor depending on its concentration and duration of action [28].

In contrast, capsaicin in the intestine floods with food but is rapidly metabolized and quickly flushes out. As a result, this could have a different pharmacodynamics of capsaicin in these two compartments.

Moreover, an anti-bacterial effect has been described against *Helicobacter pylori* and *Pseudomonas aeruginosa* [60, 61]. This means that capsaicin can potentially affect the microbiome and thus probably the whole intestinal immune system which is an interesting approach for further studies.

## Conclusion

In agreement with previous findings, our results support the thesis of a nutrition-dependent autoimmunity as well as the gut-nerve axis.

In conclusion, an early preventive oral treatment with capsaicin ameliorates the course of EAN in different biological levels from clinical course to cytokines. For autoimmune neuropathies in humans, our study opens new therapeutic approaches for a primary as well as secondary prevention in these diseases. Yet, further studies on the mechanisms of action as well as the tolerability of long-term capsaicin diet have to be carried out.

## Additional files

Additional file 1: **Figure S1.** Experimental design. Experimental overview shows (a) typical disease course in EAN; onset of symptoms between day 8 and 11 p.i.; peak of disease around day 16 p.i.; recovery phase with relief of symptoms around day 23 p.i. (b) Two start points of treatment (late preventive setting starts with day of immunization; early preventive setting imitates a long-term diet and starts 10 days before immunization). (c) Investigation overview at day 16 p.i. and day 23 p.i. (PDF 846 kb)

Additional file 2: **Figure S2.** Capsaicin protects from demyelination: electrophysiological testing in recovery phase and maximum of the disease. (A) In recovery phase of EAN (d 23 p.i.), motor nerve conduction velocity (MNCV) showed a higher nerve conduction velocity in the treated group (50 μg/d). At the maximum of disease, there was no difference between the groups. (B) At disease maximum (d16), the mean compound muscle action potential (CMAP) of the sciatic nerve is more than 50% reduced in control group as an indicator of axonal damage. (C) At disease maximum (d16) F-wave latency was significantly prolonged in control group whereas both treatment groups did not show a prolongation of F-waves. In recovery phase (d23), the group treated with 50 μg/d also showed normal F-wave latencies. (PDF 675 kb)

Additional file 3: **Figure S3.** Expression of CGRP in the sciatic nerve and regulatory T-lymphocytes in Peyer’s patches did not change. (A) Rats were daily force fed with capsaicin or rapeseed oil from day − 10 p.i. to day 16 p.i. (at expected disease maximum), sciatic nerves were isolated, and RT-PCR of calcitonin gene-related peptide CGRP was performed. Expression of CGRP did not changed significantly in RT-PCR. Mean values and SD were depicted (*p* = 0.0681, *n* = 45, *n* = 15/group, pooled data from three independent experiments). (B) FACS analyses did not show any change of CD4^+^CD25^+^FoxP3^+^ cell population in Peyer’s patches (*n* = 45, *n* = 15/group, pooled data from three independent experiments) (PDF 52 kb)

## Abbreviations

AUC: Area under the curve;
Az.: Aktenzeichen
cDNA: Complementary deoxyribonucleic acid
CFA: Completes Freud’s adjuvant
CGRP: Calcitonin gene-related peptide
CIDP: Chronic inflammatory demyelinating polyneuropathies
CJ: Campylobacter jejuni
CMAP: Compound muscle potentials
CNS: Central nervous system
DAPI: 4′,6-Diamidin-2-phenylindol
DC: Dendritic cells
EAN: Experimental autoimmune neuritis
FACS: Fluorescence-activated cell sorting
GBS: Guillain- Barré syndrome
i.p.: Intraperitoneally
MNCV: Motor nerve conduction velocity
*p* value: Probability level
p.i.: Post immunization
PBS: Phosphate buffer saline
PNS: Peripheral nervous system
PP: Peyer’s patch
RNA: Ribonucleic acid
RT-PCR: Reverse transcription polymerase chain reaction
TRPV1: Transient receptor potential channel vanilloid subfamily member 1
