# Supplementary figures and images for: Capsaicin-enriched diet ameliorates autoimmune neuritis in rats
Source: J Neuroinflammation. 2018 Apr 24;15:122. doi: 10.1186/s12974-018-1165-x (PMC5916583; doi:10.1186/s12974-018-1165-x)

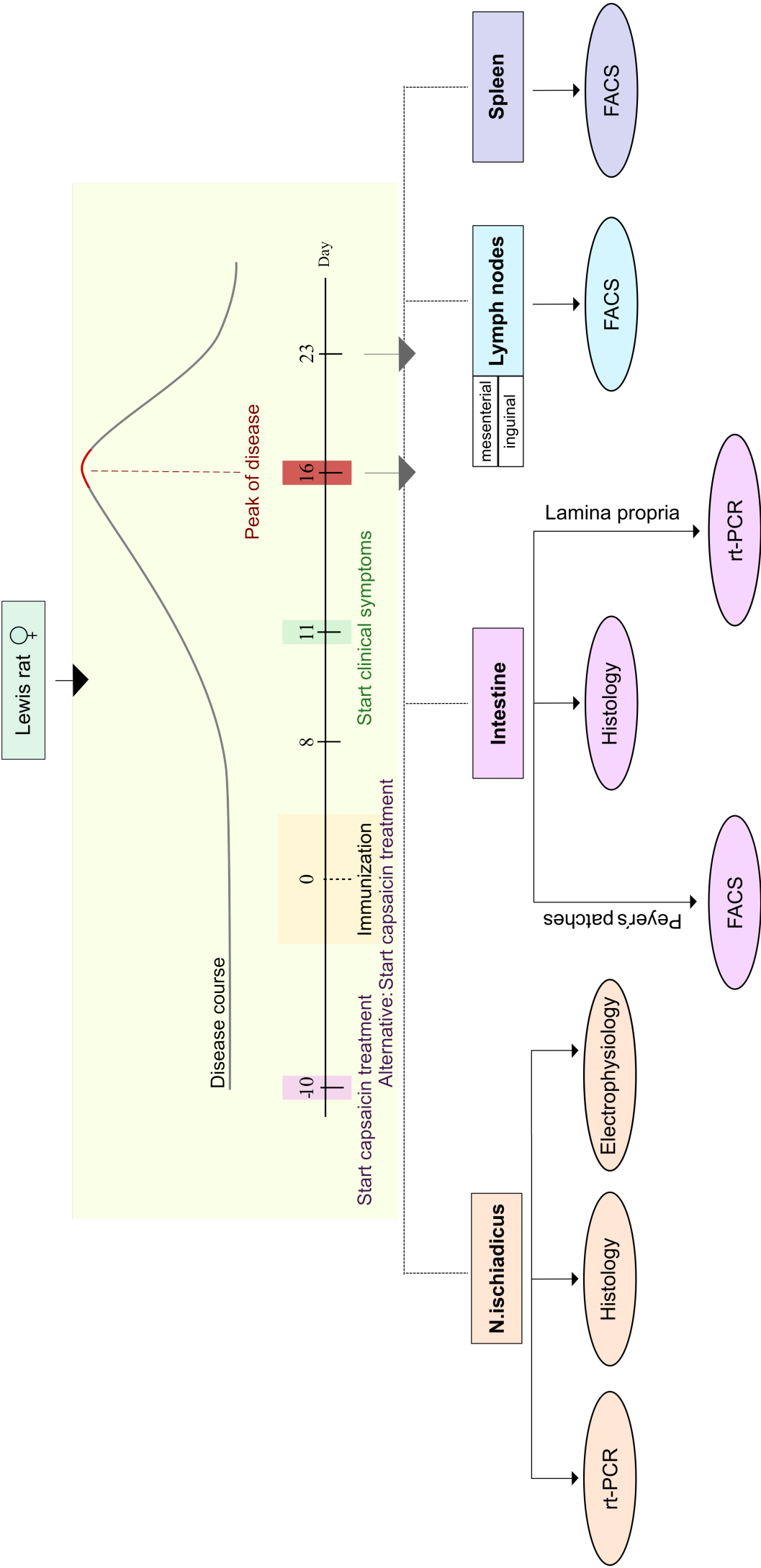

Supplement: Supplementary file 1 — Figure S1. Experimental design. Experimental overview shows (a) typical disease course in EAN; onset of symptoms between day 8 and 11 p.i.; peak of disease around day 16 p.i.; recovery phase with relief of symptoms around day 23 p.i. (b) Two start points of treatment (late preventive setting starts with day of immunization; early preventive setting imitates a long-term diet and starts 10 days before immunization). (c) Investigation overview at day 16 p.i. and day 23 p.i. (PDF 846 kb) [file 12974_2018_1165_MOESM1_ESM.pdf]

A

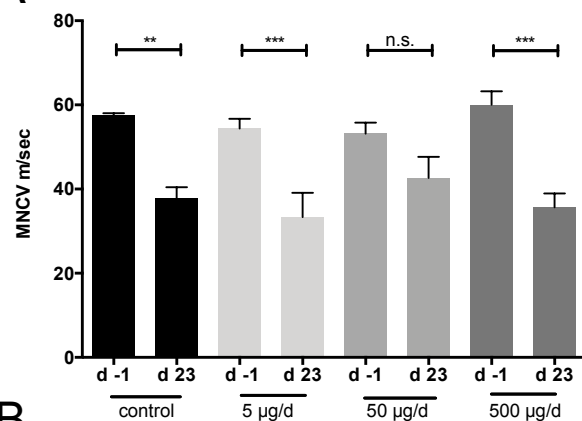

B

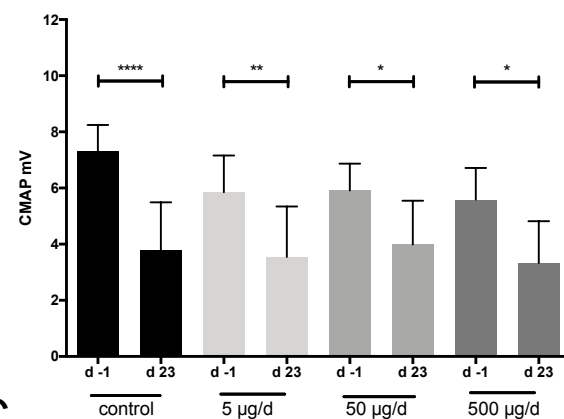

C

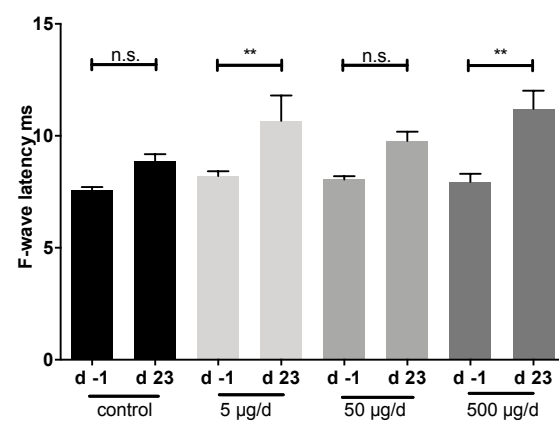

D

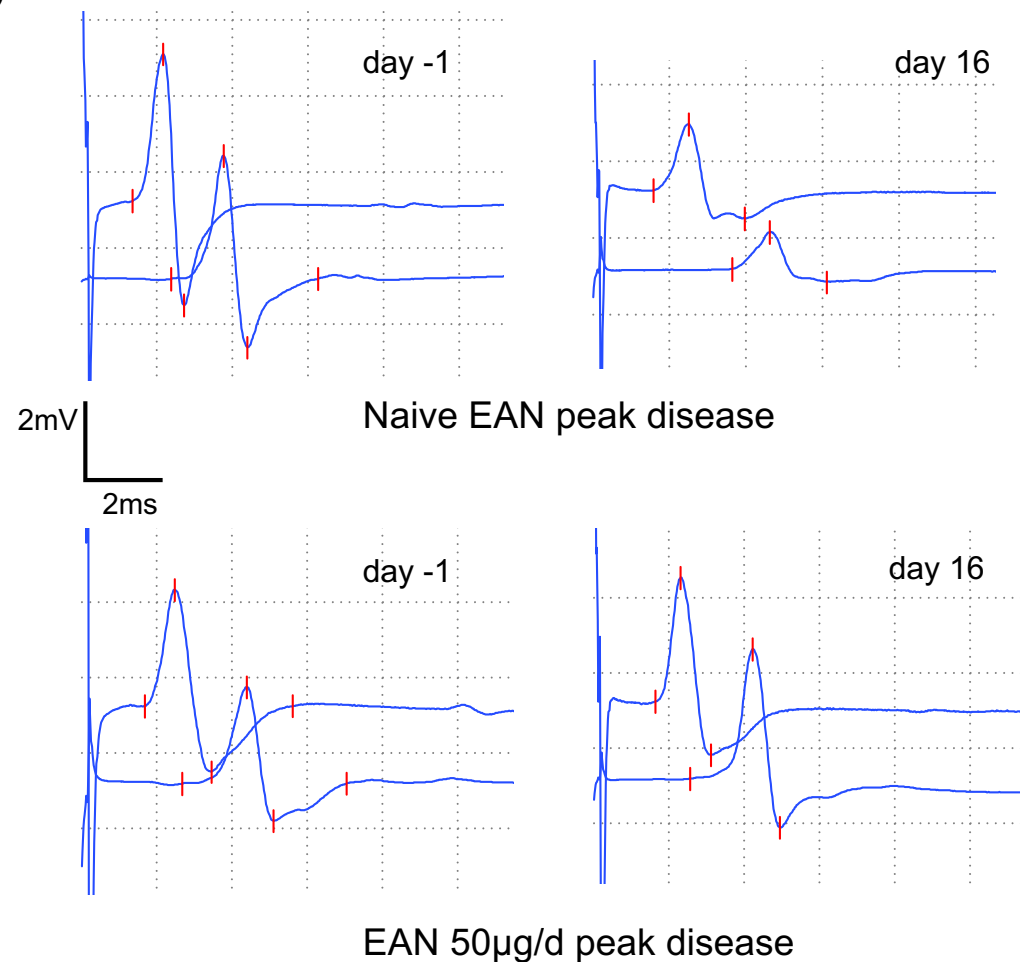

Supplement: Supplementary file 2 — Figure S2. Capsaicin protects from demyelination: electrophysiological testing in recovery phase and maximum of the disease. (A) In recovery phase of EAN (d 23 p.i.), motor nerve conduction velocity (MNCV) showed a higher nerve conduction velocity in the treated group (50 μg/d). At the maximum of disease, there was no difference between the groups. (B) At disease maximum (d16), the mean compound muscle action potential (CMAP) of the sciatic nerve is more than 50% reduced in control group as an indicator of axonal damage. (C) At disease maximum (d16) F-wave latency was significantly prolonged in control group whereas both treatment groups did not show a prolongation of F-waves. In recovery phase (d23), the group treated with 50 μg/d also showed normal F-wave latencies. (PDF 675 kb) [file 12974_2018_1165_MOESM2_ESM.pdf]

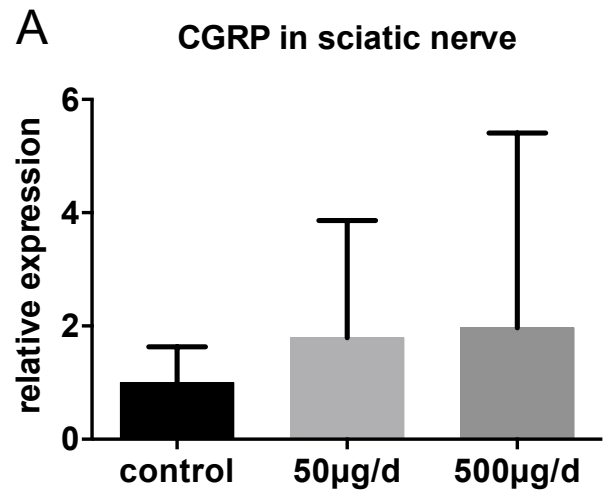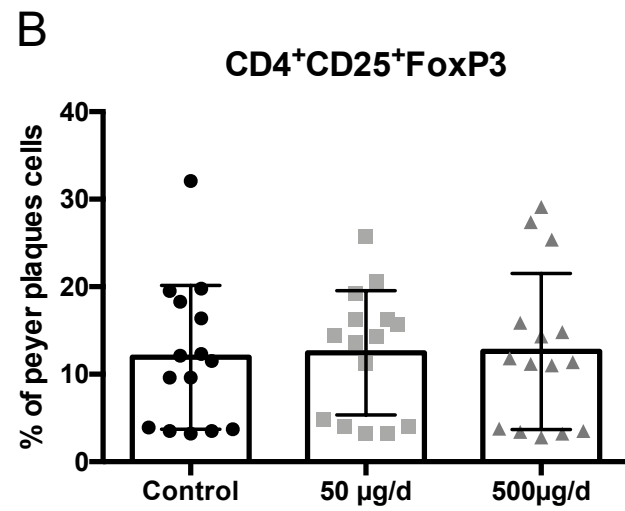

Supplement: Supplementary file 3 — Figure S3. Expression of CGRP in the sciatic nerve and regulatory T-lymphocytes in Peyer’s patches did not change. (A) Rats were daily force fed with capsaicin or rapeseed oil from day − 10 p.i. to day 16 p.i. (at expected disease maximum), sciatic nerves were isolated, and RT-PCR of calcitonin gene-related peptide CGRP was performed. Expression of CGRP did not changed significantly in RT-PCR. Mean values and SD were depicted (p = 0.0681, n = 45, n = 15/group, pooled data from three independent experiments). (B) FACS analyses did not show any change of CD4+CD25+FoxP3+ cell population in Peyer’s patches (n = 45, n = 15/group, pooled data from three independent experiments) (PDF 52 kb) [file 12974_2018_1165_MOESM3_ESM.pdf]
